# Understanding Challenges and Unmet Needs: Experiences of Latino Adult-Child Caregivers of Persons with Dementia

**DOI:** 10.1093/geroni/igaf122.3326

**Published:** 2025-12-31

**Authors:** Eunbee Kim, Hyun Jung Kim, Seunghye Hong, Jung-Ah Lee

**Affiliations:** University of California, Irvine, Irvine, California, United States; Seattle University, Seattle, Washington, United States; University of Hawaiʻi at Mānoa, Honolulu, Hawaii, United States; University of California, Irvine, Irvine, California, United States

## Abstract

The Latino/Hispanic community faces unique caregiving challenges, with family caregiving largely falling on adult children rather than spouses. As the prevalence of Alzheimer’s Disease and Related Dementias and the Latino/Hispanic population grow, the burden on these caregivers continues to increase. However, limited research has examined this role’s challenges and long-term impact, making it difficult to develop effective support. This study explores these factors among Latino/Hispanic adult-child caregivers of persons with dementia (PWD), highlighting their experiences and unmet needs. This descriptive qualitative study analyzed home-visit logs from ten adult-child caregivers, including three males, who participated in a randomized controlled trial of a 3-month home-based intervention. Key themes emerged, including struggling with financial strain, coping with persistent distress, navigating a fragmented healthcare system, and balancing competing responsibilities. Financial strain often added to caregivers’ burden, not only affecting their ability to afford care-related expenses but also increasing reliance on family or informal networks for support. Many struggled with balancing work, family obligations, and caregiving demands while facing limited support, leading to physical and emotional exhaustion. Navigating a complex healthcare system created additional barriers to securing appropriate care, with caregivers frequently encountering fragmented services, a lack of culturally competent providers, and challenges related to advanced care planning and legal matters. These challenges contribute to heightened emotional distress, increased caregiver burden, and a greater risk of depressive symptoms. Findings underscore the urgent need for targeted interventions, improved healthcare accessibility, and culturally responsive support programs to better serve Latino/Hispanic adult-child caregivers of PWD.

